# Anomalous systemic arterial supply to the left basal lung with a calcified abnormal vessel: a case report

**DOI:** 10.1186/s40792-022-01469-8

**Published:** 2022-06-22

**Authors:** Motoki Ebihara, Sakashi Fujimori, Souichiro Suzuki, Takuma Yotsumoto, Shinichiro Kikunaga, Reo Ohtsuka, Shigefumi Matsuyama

**Affiliations:** 1 Department of Thoracic Surgery, Respiratory Centre, Toranomon Hospital, 2-2-2 Toranomon, Minato-ku, Tokyo, 105-8470 Japan; 2 Department of Cardiovascular Surgery, Toranomon Hospital, 2-2-2 Toranomon, Minato-ku, Tokyo, 105-8470 Japan

**Keywords:** Congenital, Anomalous, Basal segment, Left lower lobe, Lung, Calcification, Thoracotomy

## Abstract

**Background:**

Anomalous systemic arterial supply to the normal basal segment of the left lower lobe is a congenital abnormality of the lung, frequently and is generally diagnosed at a young age. Surgery is generally recommended if symptoms such as blood sputum or fever are observed. Resection of the abnormal artery is often performed at an early age, with only few reports of surgery being performed at an older age. In addition, to the best of our knowledge, there are no reports on surgical treatment of abnormal calcified vessels to date. Herein, we have presented a case in which a calcified aberrant vessel of lung was resolved surgically.

**Case presentation:**

A 65-year-old female, previously diagnosed with anomalous systemic arterial supply to the left normal basal segment of the left lung lower lobe of lung was under observation on the basis of being asymptomatic.

The patient presented to the emergency room with the chief complaint of blood in the sputum and she was referred to our hospital for a surgery. Computed tomography showed circumferential calcification of the intima of the abnormal vessel, which might have contributed to incomplete resection of the artery if automatic sutures were used. Thus, the abnormal vessel was ligated and dissected using pledgeted 4–0 polypropylene sutures and vessel clips under open thoracotomy followed by left lower lobectomy. The patient was discharged seven days after surgery without any serious surgical complications.

**Conclusions:**

Vascular congenital anomalies of the lung are often operated at a young age presenting due to the associated symptoms. However, even if the disease is discovered incidentally and does not cause any symptoms or calcification in the aberrant artery, early surgical intervention is important due to the possibility of calcification occuring in the future. This can help minimize the degree of surgical invasion.

## Background

Anomalous systemic arterial supply to the normal basal segment of the left lower lobe of the lung is a rare congenital abnormality. This abnormality had previously been classified as a type of pulmonary sequestration (PS) by Pryce [1]. However, at present, this type of abnormality is considered as a distinct disease concept and is differentiated from PS, on the basis of absence or presence of bronchial abnormalities. This disease entity was first proposed by Painter in 1968 [2].

Surgical treatment has been reported for the management of this rare congenital abnormality. The surgical approaches that can be employed vary widely, and can include ligation, lobectomy, segmentectomy, and coiling, performed either via thoracoscopy or open thoracotomy [3–10]. Only one report of calcification of abnormal vessels treated with coiling has been published previously [11]. Moreover, surgical treatment of calcified abnormal vessels has not been reported (Table1).

**Table 1:** Review of the literature published in English to date regarding the management approach for an abnormal artery

References	Age	Gender	Diameter (mm)	Calcification	Supply	Procedures	Approach
Kuo-An et al. [15]	24	Female	Unknown	(–)	Descending thoracic aorta	Unknown	Unknown
Yamanaka et al. [10]	29	Male	30	(–)	Descending thoracic aorta	Ligation	Posterolateral incision
	30	Male	9	(–)	Descending thoracic aorta	The proximal end was oversewn with 3–0 polypropylene End-to-side anastomosis between the anomalous artery and the inferior side of the pulmonary artery was performed with 5–0 polypropylene without resection	Posterolateral incision
	46	Male	20	(–)	Descending thoracic aorta	Ligation	Posterolateral incision
	68	Female	20	(–)	Descending thoracic aorta	Ligation	Posterolateral incision
Higuchi et al. [5]	58	Male	25	(–)	Descending thoracic aorta	Autostapler	Thoracotomy
Akiba et al. [3]	55	Female	Unknown	(–)	Descending thoracic aorta	Autostapler	VATS
Singhi [8]	30 days	Baby	Unknown	(–)	Descending thoracic aorta	Ligation	Thoracotomy
Mori et al. [7]	49	Male	10	(–)	Descending thoracic aorta	Autostapler	VATS
	22	Male	15	(–)	Descending thoracic aorta	Autostapler	VATS → thoracotomy
	54	Male	16	(–)	Descending thoracic aorta	Autostapler	VATS
	25	Female	15	(–)	Descending thoracic aorta	Autostapler	VATS
Albertini et al. [4]	21	Male	Unknown	(–)	Descending thoracic aorta	Ligation	Thoracotomy
Makino et al. [6]	33	Male	7,12	(–)	Descending thoracic aorta	Autostapler	VATS
Utsumi et al. [16]	42	Male	33	(–)	Descending thoracic aorta	Autostapler	Thoracotomy

*VATS* video-assisted thoracic surgery

Herein, we report a case of a calcified abnormal vessel of the lung, safely using reinforced ligation.

## Case presentation

A 65-year-old woman was diagnosed with anomalous systemic arterial supply to the normal basal segment of the lung in her early forties. As she was asymptomatic, she had been under observation only since the time of her diagnosis. Her medical history included hypertension; she was found to have hypertension two years ago when she was admitted to a previous medical emergency for hypertension.

She reported to the emergency room of her previous doctor with the chief complaint of blood in her sputum. After a computed tomography (CT) scan, the anomalous systemic arterial supply to the normal basal segment was suspected as the cause of the presenting symptom, and surgery was considered necessary. Thereafter, the patient was referred to our hospital for surgical treatment.

**Fig. 1:**
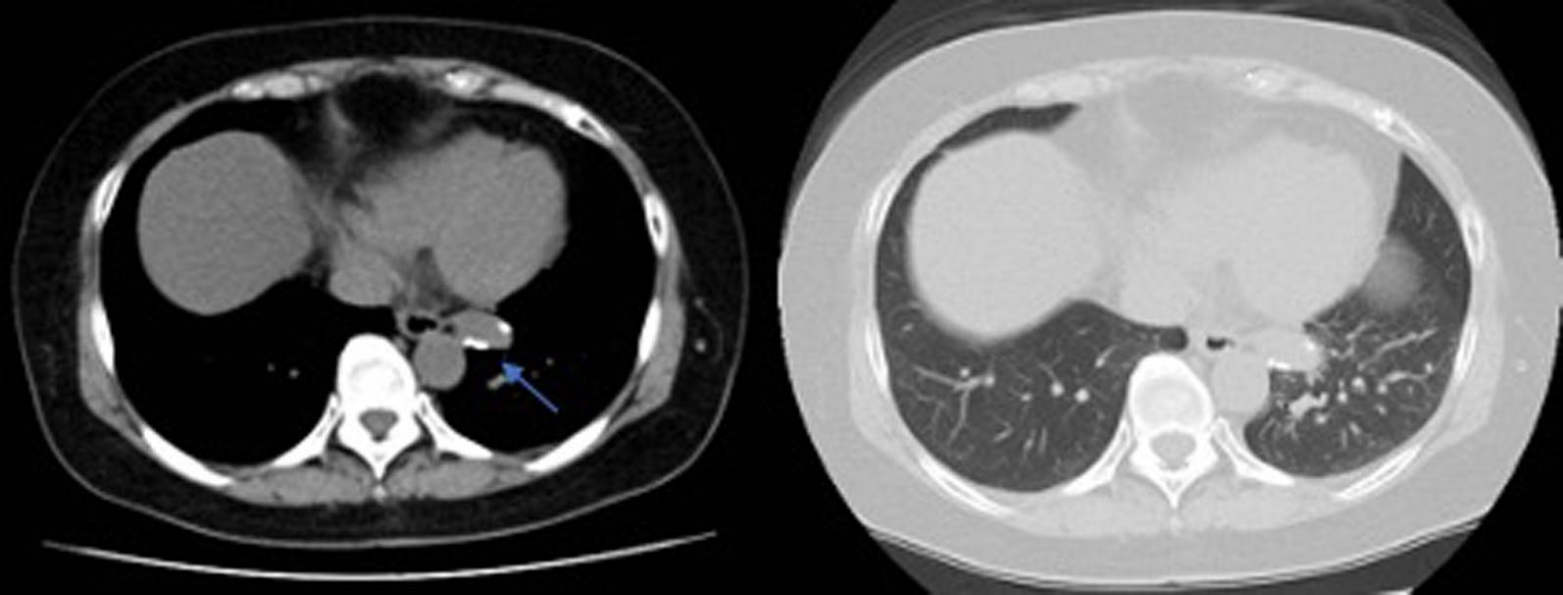
CT of the chest showing the abnormal artery with calcification(arrow)

**Fig. 2:**
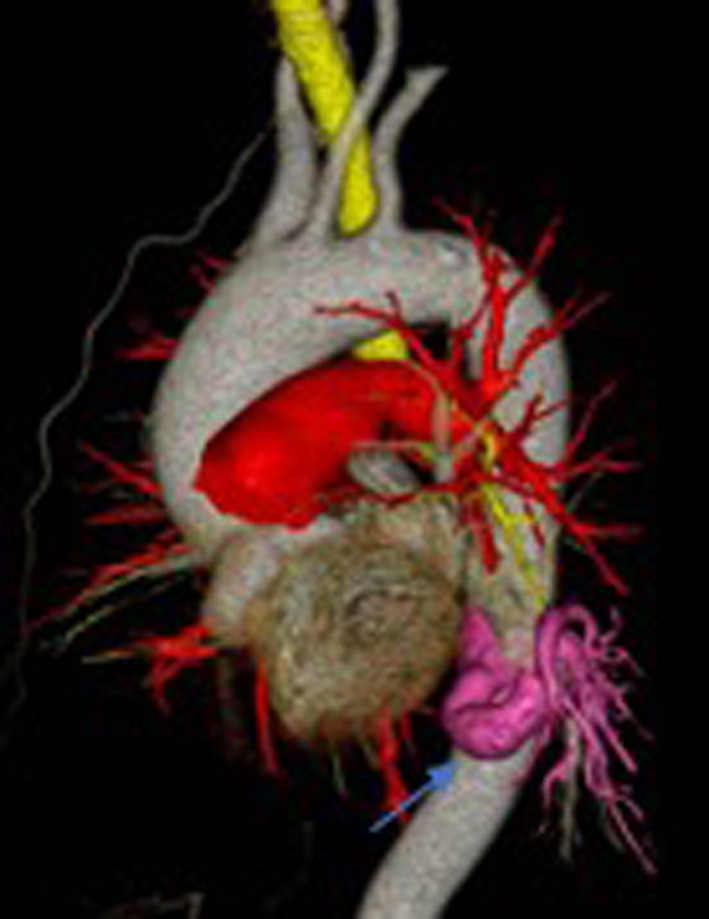
Three-dimensional CT image of the aberrant artery arising from the descending aorta(arrow)

CT images revealed a calcified aberrant artery with maximum diameter of 11 mm at the root. The vessel originated from the descending aorta at the level of the eighth to ninth dorsal ribs (Figs. 1 and 2). Thickening and calcification of the vessel wall was observed mainly at the root. There was no abnormality in the pulmonary vein. However, a shadow suspicious of pneumonia was also present in S6 beyond the pulmonary vein and between the S6 and the basal segment. After discussions with the vascular surgeons open chest surgery was decided to be the safer option as the use of automatic sutures to treat the calcified vessel carries the risk of an incomplete resection. The decision was then made to perform a left lower lobectomy, as the image of pneumonia was also present in the S6.

Pulmonary function examination showed a forced expiratory volume per second of 1.69L (80.1% of the predicted volume) and a forced vital capacity of 2.11L (77% of the predicted volume). Echocardiography showed good cardiac contractility with an ejection fraction of 65%. The patient’s TR max was 2.4 m/s, TAPSE was 10.2 mm, and TRPG was 23.2 mmHg.

**Fig. 3:**
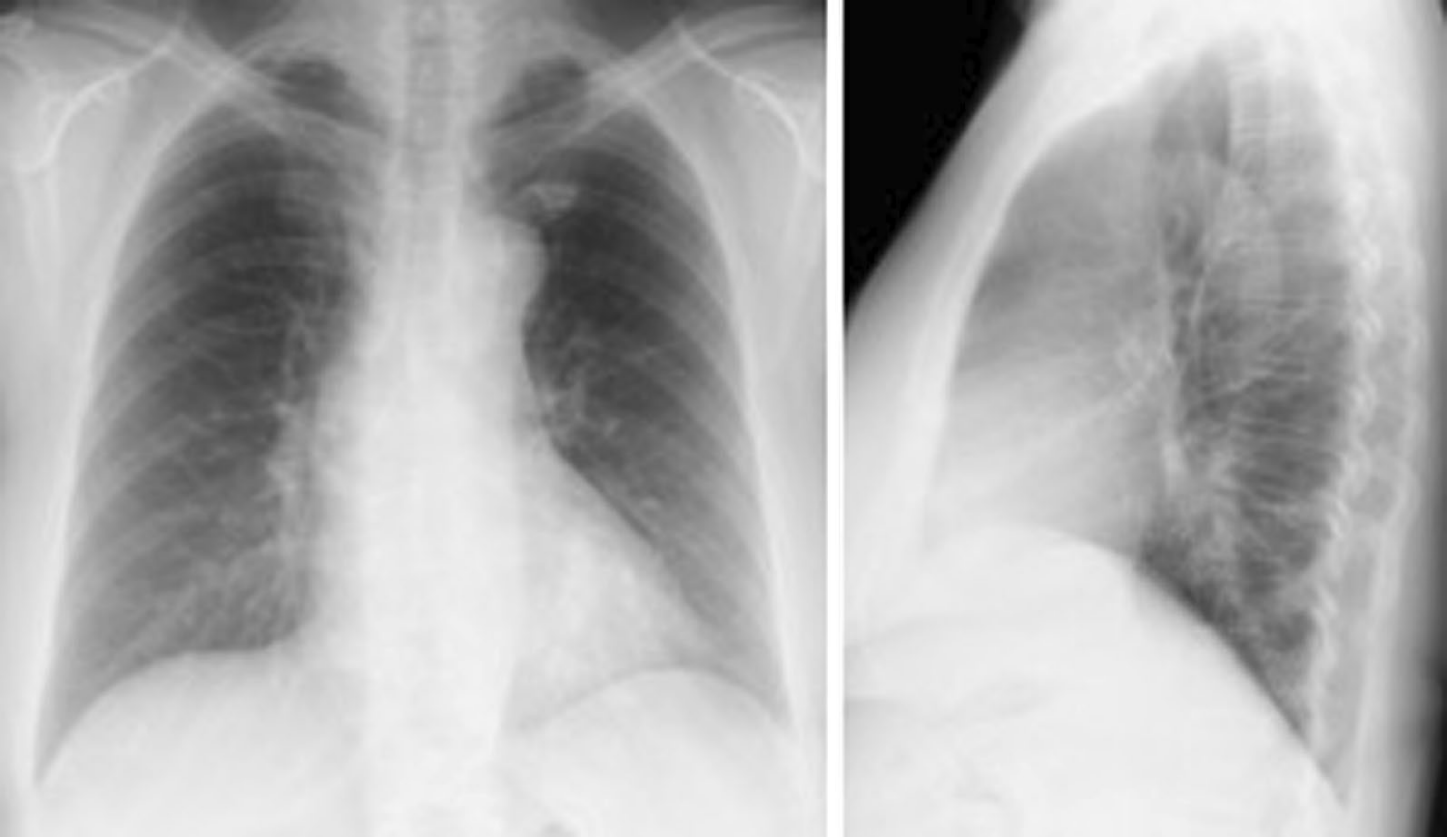
Chest x-ray taken when the patient was admitted to the hospital

**Fig. 4:**
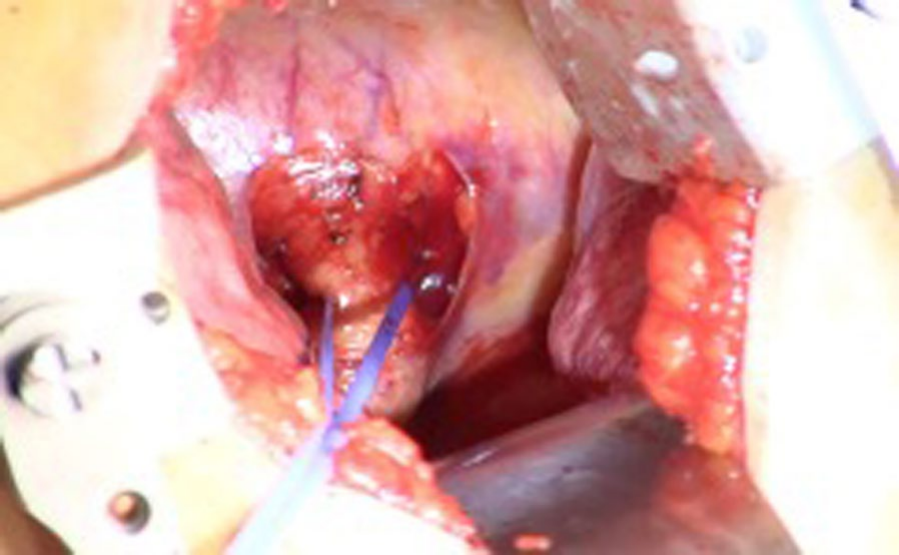
Picture showing the thoracic artery supplying blood to the aberrant artery

**Fig. 5:**
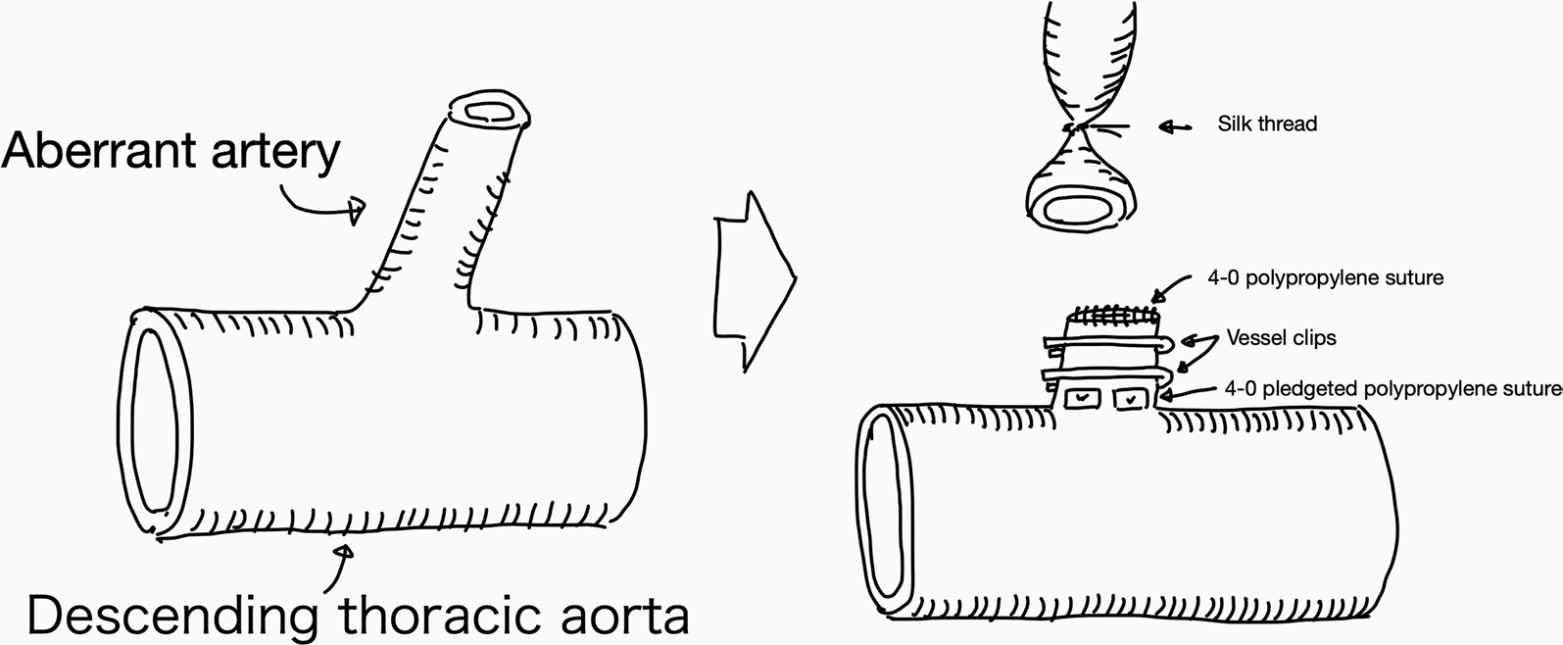
Illustration of the treatment of the aberrant artery

We performed a left lower lobectomy with aberrant artery excision. First, the patient was placed in the left lateral recumbent position under general anesthesia (Fig. 3). The thoracic cavity was approached from the sixth intercostal space and the aberrant artery of about 1.5 cm in diameter was detected to be originating from the descending aorta (Fig. 4). Macroscopic changes between the normal lung parenchyma and the parenchyma perfused by the aberrant artery were observed, such as dilated capillaries. The origin of the aberrant vessel was sutured with two pledgeted 4–0 polypropylene sutures. In addition, two clips were placed in order to close off the central site of the vessel suture. The peripheral side was then ligated with a silk thread. The abnormal artery was dissected between the pledgeted 4–0 polypropylene sutures and the silk thread. Thereafter, a 4–0 polypropylene suture was used to close the end of the vessel. (Fig. 5) Then, the lower pulmonary vein and pulmonary artery of the lower lobe were resected in the same order. Side clamping of the descending aorta was not necessary. After this, the pathological specimen was removed by separating the left lower lobar bronchus. The operation time was 162 min with a total blood loss of 50 mL. On post-operative day one, the drain was removed and the patient was discharged without any complication on post-operative day seven.

Histopathological examination of the surgical specimen revealed elastic type artery exhibiting intimal thickening and lumen dilation with calcification. On its peripheral side, some atheroma formation was also observed.

## Discussion

Anomalous systemic arterial supply to the left basal lung is a relatively rare disease. This pathology was classified by Pryce et al. as type I sequestration, with no abnormalities in the bronchial branches [1].

Patients with this anomaly often need surgery because of the risk of hemoptysis due to pulmonary hypertension associated with left-to-left shunt [12]. Surgery has been performed on patients of a wide age range. Both open thoracotomy and thoracoscopic approaches are used to perform the surgery, based on the individual requirements of each case. As for calcification of the abnormal vessel, only one case has been reported to date, in which coiling was selected as the method of treatment[11].

In the past, we had resected aberrant arteries of this disease using autosutures under video-assisted thoracoscopic surgery (VATS) in all cases presented with this disease. However, in the present case, we decided to handle the aberrant artery using ligation with clips under thoracotomy, based on the concern that incomplete dissection by automatic suture machine due to the dense calcification could lead to uncontrollable bleeding from the dissected end due to the dense calcification. Hence we rigorously dissected the vessel using two 4–0 polypropylene sutures and two vessel clips and were able to complete the surgery without bleeding.

Another option in such cases is to treat the vessel on the peripheral side of the calcification with an automatic suture. However, there is a risk of aneurysm formation in the sutured area due to pressure from the left ventricular system[13]. The first postoperative plain CT scan showed no aneurysm. Coil embolization was not chosen as a treatment modality because of the risk of inadequate embolization or embolism in vessels larger than 10 mm[14]. In addition, the technique of ligation only was not chosen because the preoperative CT showed evidence of pneumonia.

It seemed to occur over time with advancing age under the condition of the left ventricular system. The only case of calcification of an aberrant artery reported so far occurred in a 53 year old patient. The lack of other reports of similar calcification can be attributed to the fact that surgery is often performed for these patients at a young age in these pationts.

Considering the above, it can be inferred that in asymptomatic cases with accidental detection of anomalous arterial supply, patients are likely to be kept on regular follow up. In the present case, however, the calcification of the abnormal blood vessel forced us to perform open thoracotomy to treat the vessel. Calcification of abnormal blood vessels can complicate VATS and necessitate invasive surgery. Therefore, even if there are no symptoms at the time of diagnosis, given the possibility of calcification occurring in the future, surgical options should still be considered at the time of diagnosis.

The present paper describes only one case; it is therefore necessary to evaluate a larger number of cases. In addition, the reported patient’s previous CTs were performed at other hospitals and could not be obtained this time. Thus, it was not possible to assess the course of changes in the calcified lesions, and it remains debatable whether abnormal vessels do indeed calcify with age.

## Conclusions

Early surgery is recommended for the management of anomalous systemic arterial supply of the lung, considering that calcification of abnormal blood vessels may occur with age, requiring more invasive procedures for treatment in the future.

## Data Availability

The dataset supporting the conclusions of this article is included within the article.

## Abbreviations

CT: Computed tomography
PS: Pulmonary sequestration
TAPSE: Tricuspid annular plane systolic excursion
TR: Tricuspid regurgitation
TRPG: Tricuspid regurgitation peak gradient
VATS: Video-assisted thoracoscopic surgery
